# UBE2C enhances temozolomide resistance by regulating the expression of p53 to induce aerobic glycolysis in glioma: UBE2C enhances the TMZ resistance in glioma

**DOI:** 10.3724/abbs.2024033

**Published:** 2024-04-17

**Authors:** Kun Zhou, Dexin Wang, Xiaolin Du, Xia Feng, Xiaoxi Zhu, Cheng Wang

**Affiliations:** 1 Department of Neurosurgery the Jinyang Hospital Affiliated to Guizhou Medical University Guiyang 550084 China; 2 Department of Sleep Medicine the Second People’s Hospital of Guizhou Province Guiyang 550084 China; 3 Key Laboratory of Cell Engineering of Guizhou Province Affiliated Hospital of Zunyi Medical University Zunyi 563000 China

**Keywords:** UBE2C, p53, TMZ resistance, aerobic glycolysis, glioma

## Abstract

UBE2C is overexpressed in gliomas, and its overexpression has been reported to be correlated with the drug resistance of gliomas to some extent. In this study, we explore the role of UBE2C in regulating temozolomide (TMZ) resistance in glioma and investigate the underlying mechanisms involved. Twenty normal brain tissues and 100 glioma tissues from 50 TMZ-resistant patients and 50 TMZ-sensitive patients are included in this study. TMZ-resistant cell lines are constructed to explore the role of UBE2C in regulating glioma cell viability and TMZ resistance. Our results show that both the mRNA and protein levels of UBE2C are significantly elevated in the brain tissues of glioma patients, especially in those of TMZ-resistant patients. Consistently, UBE2C expression is markedly upregulated in TMZ-resistant cell lines. Overexpression of UBE2C rescues glioma cells from TMZ-mediated apoptosis and enhances cell viability. In contrast, downregulation of UBE2C expression further enhances TMZ function, increases cell apoptosis and decreases cell viability. Mechanistically, UBE2C overexpression decreases p53 expression and enhances aerobic glycolysis level by increasing ATP level, lactate production, and glucose uptake. Downregulation of p53 level abolishes the role of UBE2C downregulation in inhibiting TMZ resistance and aerobic glycolysis in glioma cells. Moreover, an animal assay confirms that downregulation of UBE2C expression further suppresses tumor growth in the context of TMZ treatment. Collectively, this study reveals that downregulation of UBE2C expression enhances the sensitivity of glioma cells to TMZ by regulating the expression of p53 to inhibit aerobic glycolysis.

## Introduction

Glioma is a common malignant intracranial tumor that accounts for >70% of all brain tumors in adults and is characterized by high invasiveness, high heterogeneity, high recurrence rate, and poor prognosis [ 1, 2]. The etiology of gliomas is not well established; however, genetic predisposition, exposure to ionizing radiation or electromagnetic fields, traumatic brain injury, and the ingestion of nitroso compounds are considered as important risk factors [1]. Surgical resection combined with postoperative adjuvant radiotherapy and alkylating agents such as temozolomide (TMZ) is the main treatment option for glioma, which significantly improves the overall survival (OS) and progression-free survival (PFS) of glioma patients, but the prognosis remains unsatisfactory [ 3, 4]. Additionally, most patients with high-grade glioma (HGG) relapse because of TMZ resistance [4]. Hence, overcoming the TMZ resistance of gliomas is crucial.

Ubiquitin-binding enzymes (E2) are members of a structurally associated protein family that mediates ubiquitin-dependent proteolysis and is implicated in multiple biological processes, *e*. *g*., cell cycle, cell proliferation, cell death, and signal transduction [5]. Ubiquitin-conjugating enzyme E2C (UBE2C) belongs to the E2 family and is upregulated in many types of tumors, such as ovarian cancer, breast cancer, pancreatic cancer and glioma, where it serves as an oncogene [ 6‒ 9]. For example, Ma *et al*. [10] reported that UBE2C expression was elevated in glioma, which predicted a worse prognosis. Alafate *et al*. [11] reported that UBE2C expression was relevant to the treatment resistance of gliomas, suggesting that UBE2C might play a role in modulating chemotherapy resistance in gliomas. However, the role of UBE2C in modulating TMZ resistance in glioma and the related mechanisms have not yet been reported.

Increasing evidence has demonstrated that dysregulated glycolysis is closely implicated in tumor cell growth, apoptosis, and drug resistance in many types of cancers [ 12, 13], including glioma [14]. Additionally, previous studies have shown that the tumor suppressor p53 plays a role in modulating glycolysis [15]. Notably, it has been reported that UBE2C downregulates p53 expression in endometrial cancer [16], indicating that UBE2C may be involved in regulating the drug resistance of glioma through modulating the p53/glycolysis axis.

In the present study, both *in vivo* and *in vitro* experiments were carried out in this study to explore the role of UBE2C in regulating TMZ resistance in glioma and to elucidate the underlying mechanisms involved.

## Materials and Methods

### Patient samples

Twenty normal brain tissue samples and 100 glioma tissue samples collected from 50 TMZ-resistant and 50 TMZ-sensitive patients were included in this study. All 100 patients with glioma received TMZ following surgery. Patients who presented with progressive or relapsed disease within 6 months after TMZ treatment were considered as TMZ-resistant patients, while patients who progressed or relapsed >6 months or without recurrence following TMZ treatment were considered as TMZ-sensitive patients, as previously described [17]. The clinical information is shown in Table 1. Informed consent was obtained from each participant, and this study was approved by the Ethics Committee of the Jinyang Hospital Affiliated to Guizhou Medical University.

**Table:** **Table 1** Clinicopathological features of 100 glioma patients

Variable	Group		Number
Gender	Female		45 (45%)
	Male		55 (55%)
Age (years, mean±SD)			52.5±15.7
Location	Supratentorial		88 (88%)
	Subtentorial		12 (12%)
Tumor number	Single		85 (85%)
	Multiple		15 (15%)
WHO grade	I‒II		16 (16%)
	III‒IV		84 (84%)

### Generation of TMZ-resistant cell lines

U-118 MG and LN-18, two human glioma cell lines, were obtained from ATCC (Manassas, USA) and cultivated in Dulbecco’s modified Eagle’s medium supplemented with 10% fetal bovine serum (FBS) and 1% (v/v) penicillin/streptomycin (TransGen Biotech, Beijing, China). The U-118 MG cell line was authenticated by STR (Short Tandem Repeat) technology (Abiowell, Changsha, China). To construct TMZ-resistant cell lines (U-118 MG/TMZ and LN-18/TMZ), U-118 MG and LN-18 cells were grown in increasing concentrations of TMZ (MedChemExpress, Shanghai, China) for 6 months until they were resistant to 50 μg/mL TMZ. All cells were cultured at 37°C with 5% CO _2_. Cell culture medium and FBS were obtained from Thermo Fisher Scientific (Waltham, USA). U-118 MG/TMZ and LN-18/TMZ cells were treated with 60 μg/mL TMZ for subsequent *in vitro* assays.

### Modification of gene expression

UBE2C overexpression lentiviral vectors (OE-UBC2C; cat No. RC208741L3V) and UBE2C/p53 knockdown lentiviral vectors (sh-UBE2C, cat. TL318745V; sh-p53, cat No. TL320558V), as well as negative control vectors (OE-NC, sh-NC), were obtained from Origene (Beijing, China). These lentiviruses were introduced into cells with the aid of 6 μg/mL polybrene. sh-UBE2C-infected cells were cultured with 7 μg/mL puromycin for 14 days to construct the stable cell lines used in the animal experiments.

### Quantitative reverse transcription-PCR (qRT-PCR)

Total RNA was extracted using Trizol reagent (Invitrogen, Carlsbad, USA), followed by cDNA synthesis using the PrimeScript RT Master Mix kit (RR036A; TaKaRa, Dalian, China) according to the manufacturer’s instructions. After that, 2×SYBR Green PCR Master Mix (Solarbio, Beijing, China) was used for PCR on a 7500 Real-Time PCR System (Applied Biosystems, Foster City, USA). The primers used in this study were as follows: *UBE2C* (forward) 5′-CGAGTTCCTGTCTCTCTGCC-3′, *UBE2C* (reverse) 5′-TGCTCCATGGATGGTCCCTA-3′; and *β-actin* (forward) 5′-ACAGAGCCTCGCCTTTGCC-3′, *β-actin* (reverse) 5′-TGGGGTACTTCAGGGTGAGG-3′.

### Western blot analysis

Total protein was isolated from tissues and cells using RIPA lysis buffer (Solarbio) supplemented with 1% protease inhibitor (Solarbio). Subsequently, 20 μg of protein collected from each group was added to a 10% sodium dodecyl sulfate-polyacrylamide gel, followed by separation by electrophoresis. The proteins were then transferred to polyvinylidene difluoride membranes (Millipore, Billerica, USA). Next, the membranes were blocked with 5% non-fat milk at room temperature for 60 min, followed by incubation with primary antibodies at 4°C for 15 h, including anti-β-actin [1:2000; #4970; Cell Signaling Technology (CST), Beverly, USA], anti-UBE2C (1:1000; ab252940; Abcam, Cambridge, USA), anti-Bcl-2 (1:1000; 15071; CST), anti-Bax (1:1000; 2744; CST), anti-cleaved caspase-3 (1:1000; 9664; CST), and anti-p53 (1:10,000 dilution; ab32389; Abcam) antibodies. Then, the membranes were incubated with horseradish peroxidase (HRP)-conjugated secondary antibodies for 6 h at 4°C. ProfiBlot-48 (Tecan, Beijing, China) and Enhanced chemiluminescence (ECL) reagents (Millipore) were used for protein imaging, followed by protein quantification using ImageJ software.

### Glycolysis assessment

Glycolysis was assessed by detecting ATP level, lactate production, and glucose consumption using an ATP assay kit, a lactate assay kit, and a glucose assay kit (Abcam), respectively, according to the manufacturer’s instructions. Relative levels were assessed by normalization to the control group.

### Cell Counting Kit-8 (CCK-8) assay

The half maximal inhibitory concentration (IC _50_) of TMZ and cell proliferation were investigated via a CCK-8 assay. For the determination of IC _50_ values of TMZ, 1×10 ^4^ cells were seeded into 96-well plates and exposed to different concentrations of TMZ (0, 0.5, 1, 5, 10, 25, 50, 100, 200, 500 or 1000 μg/mL) for 24 h. Next, 10 μL of CCK-8 reagent (Beyotime, Beijing, China) was added to each well. After the cells were cultured for 3 h, the optical density (OD) was detected at 450 nm using a microplate reader (Fisherbrand™ accuSkan™ GO UV/Vis; Thermo Fisher Scientific). Cell viability was normalized to that of the control group (0 μg/mL TMZ), and the IC _50_ value of TMZ was determined according to the viability curves.

For cell proliferation assessment, cells in each well of 96-well plates were incubated with 10% (v/v) CCK-8 solution for 4 h at 37°C. Subsequently, a spectrophotometer (Fisherbrand™ accuSkan™ GO UV/Vis) was used to measure the OD values (450 nm) of each well.

### Flow cytometry

After 48 h of treatment, the cell apoptosis for each group was tested using the FITC Annexin V Apoptosis Detection kit (BD Biosciences, Franklin Lakes,, USA) according to the manufacturer’s instructions. Cell apoptosis rates were analyzed using FlowJo software.

### Terminal deoxynucleotidyl transferase (TdT) dUTP nick-end labelling (TUNEL)

Lentivirus-infected and/or TMZ-treated cells were collected, fixed with 4% paraformaldehyde for 15 min, and permeabilized in 0.25% Triton‐X 100 for 20 min. Then, the TUNEL assay was performed using a TUNEL kit (Roche, Shanghai, China) according to the manufacturer’s protocol. Briefly, the cells were subjected to a Click-iT reaction followed by incubation with terminal deoxynucleotidyl transferase (TdT) for 45 min at 37°C. The nuclei were stained with hematoxylin. Cell staining was visualized using a Zeiss LSM710 confocal microscope (Carl Zeiss, Wetzlar, Germany).

### Xenograft experiments

Fifteen 6-week-old BALB/c nude mice (20±2 g; Chinese Academy of Sciences, Shanghai, China) were used in this experiment. Mice were housed at 25°C with 55% humidity under a 12-h light/12-h dark cycle with *ad libitum* access to water and food. For the experimental procedure, mice were subcutaneously injected with 2×10 ^6^ sh-NC-infected or sh-UBE2C-infected U-118 MG/TMZ cells in the right flank, with 5 and 10 mice in each treatment, respectively. Seven days later, the mice were administered with 20 mg/kg TMZ or an equal volume of phosphate buffer solution (PBS) via intraperitoneal injection twice a week. Mouse health and behavior were monitored every 3 days. Mice were euthanized 28 days after the initial administration of TMZ/PBS or when the tumor diameter exceeded 1 cm. In this study, all mice were euthanized 28 days after the initial administration of TMZ. Following the administration of vaporized isoflurane (inhaled) for anesthesia at a concentration of 2.0% for induction and 1.0% for maintenance, the mice were euthanized through cervical dislocation. The tumors were harvested for further analysis, including immunohistochemistry (IHC) staining, western blot analysis, and TUNEL. The tumor volume (V) was calculated as follows: V=length×width ^2^/2.

### Immunohistochemistry (IHC)

Tissue sections were deparaffinized and subjected to antigen retrieval, blocked with 1% BSA and incubated with an anti-UBE2C antibody (1:500; ab252940; Abcam) or anti-p53 antibody (1:100; ab32389; Abcam) overnight at 4°C. After that, the sections were incubated with an HRP-labeled secondary antibody (18653; CST) for 1 h at room temperature. The expression levels of UBE2C and p53 were scored by the area and intensity of the staining. Specifically, the stained area in each region of interest was scored as follows: 0 for a percentage<5%, 1 for 5%‒25%, 2 for 25%‒50%, 3 for 50%‒75%, and 4 for >75%. The staining intensity was scored as 0, 1, 2 or 3 for negative (no staining), mild (weak), intermediate (distinct) or intense (strong) staining, respectively. The staining intensity and percentage of stained area were multiplied to obtain a weighted score [17].

### Statistical analysis

The experiments in this study were independently performed three times. Student’s *t* test and one-way ANOVA with Tukey’s test were applied for difference analysis using SPSS 22.0 software (IBM, Armonk, USA). The Kaplan-Meier method with the log-rank test was used for survival analysis. Statistical significance was set at *P*<0.05.

## Results

### UBE2C expression is elevated in glioma patients with TMZ resistance

**Figure 1:**
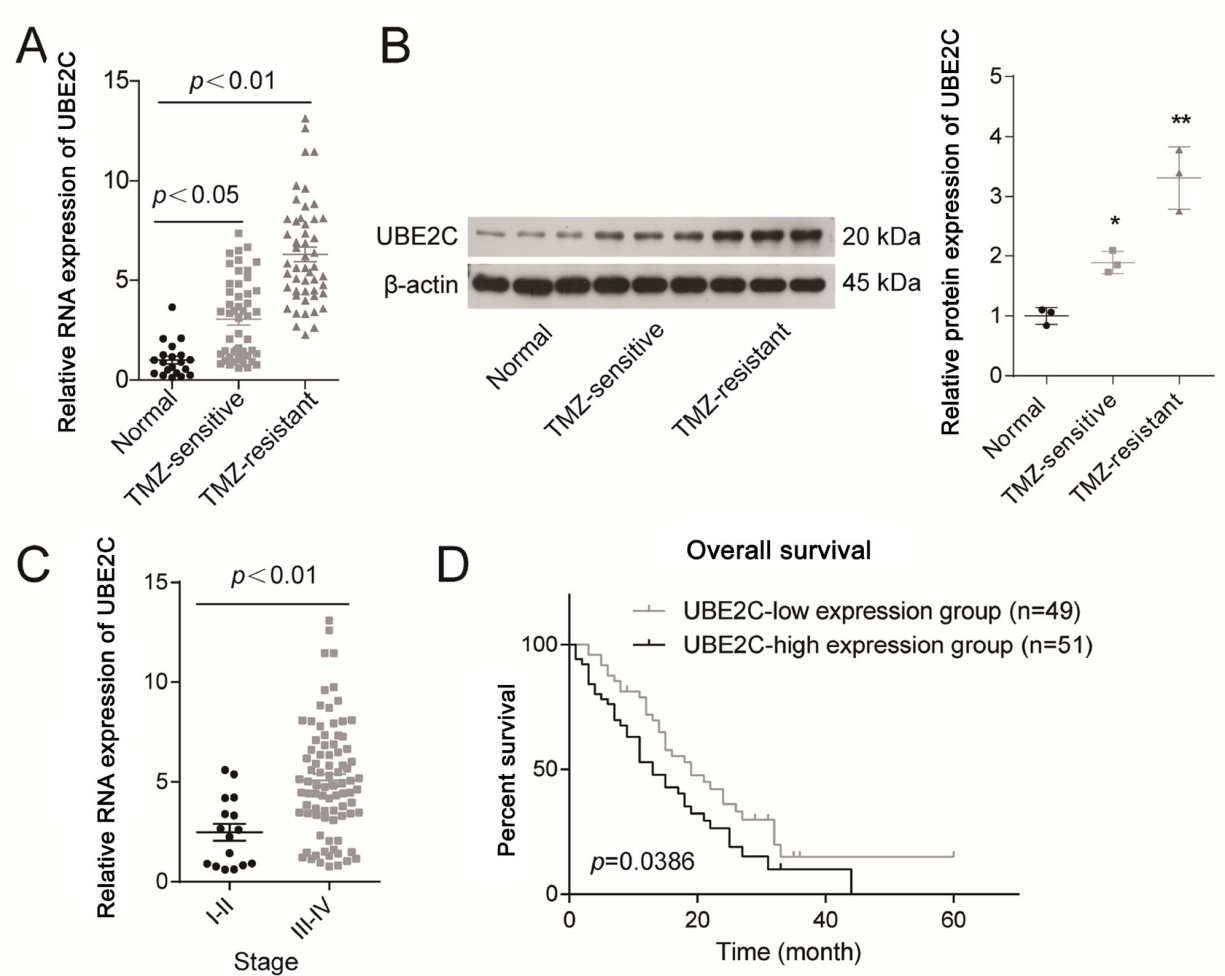
Figure 1 UBE2C expression is elevated in glioma patients who were resistant to TMZ, and high UBE2C expression was linked to poor prognosis (A,B) The expression of UBE2C at the (A) mRNA and (B) protein levels was detected in normal brain tissues and brain tissues from TMZ-resistant and TMZ-sensitive tissues by qRT-PCR and western blot analysis. (C) The mRNA levels of UBE2C in I‒II and III‒IV gliomas were assessed. (D) Kaplan-Meier curves were generated to evaluate the effect of UBE2C expression levels on glioma patients’ overall survival. *P<0.05, **P<0.01 vs normal group.

To reveal the role of UBE2C in regulating TMZ resistance in gliomas, we first assessed its expression levels in the brain tissues of TMZ-resistant and TMZ-sensitive glioma patients, as well as in normal brain tissues. Compared with those in normal brain tissues, the mRNA and protein levels of UBE2C were elevated approximately 1-fold and 0.8-fold, respectively, in the brain tissues of glioma patients who were sensitive to TMZ but 5-fold and 2.2-fold, respectively, in patients with TMZ resistance ( Figure 1A,B). Additionally, the mRNA levels in patients with I‒II gliomas were significantly greater than those in patients with III‒IV gliomas ( Figure 1C). In addition, we found that the overall survival rate of patients with high UBE2C expression (≥the median expression level of UBE2C between the patients) was significantly lower than that of patients with low UBE2C expression (<the median expression level of UBE2C between the patients), with 50% overall survival time of approximately 13 months and 20 months for the UBE2C high expression and low-expression groups, respectively ( Figure 1D). These results indicate that UBE2C is more highly expressed in glioma patients with TMZ resistance than in patients with TMZ sensitivity and may play a role in regulating TMZ resistance in gliomas.

### Upregulation of UBE2C enhances cell growth and drug resistance in glioma

**Figure 2:**
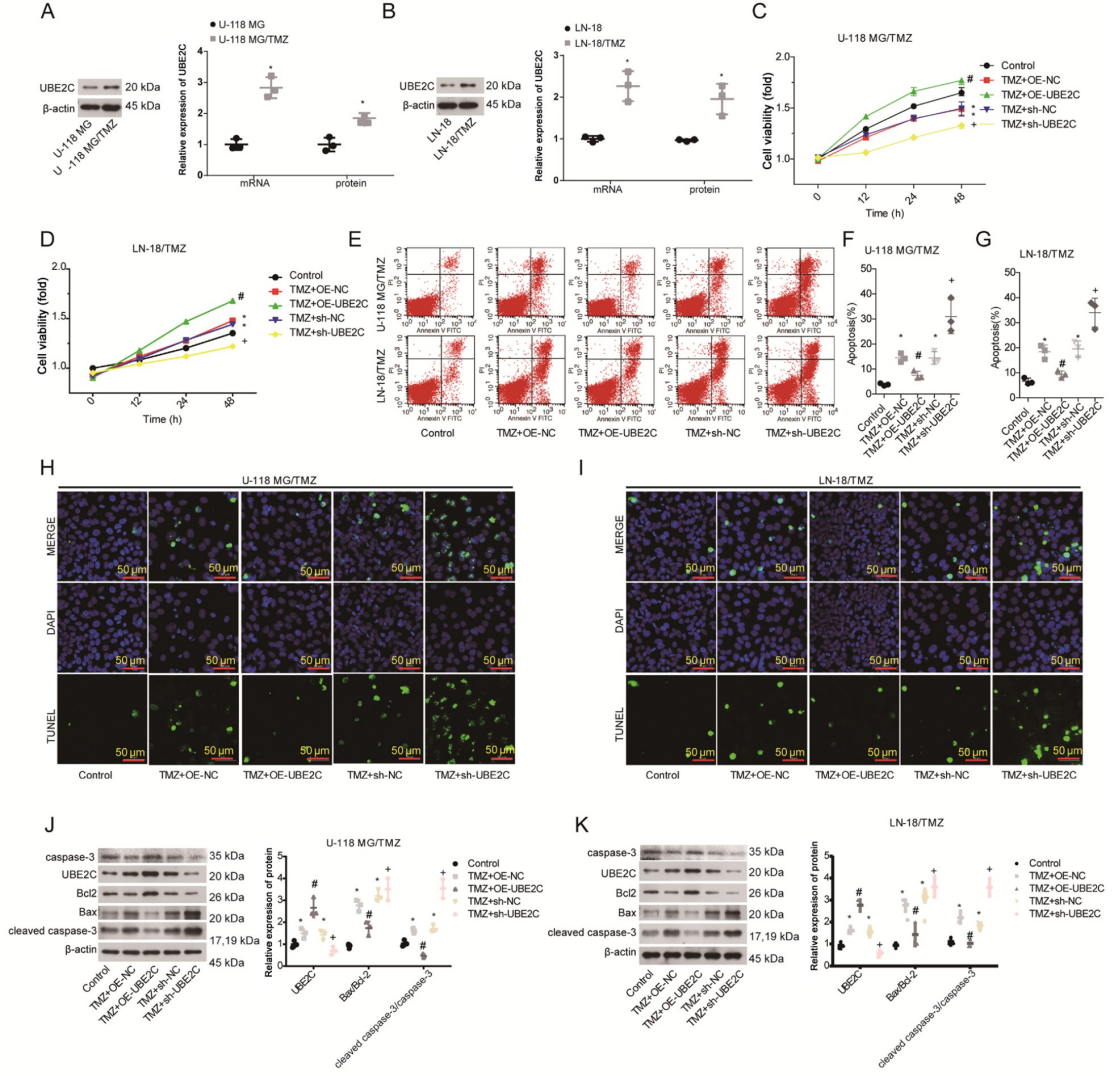
Figure 2 Upregulation of UBE2C enhances cell growth and drug resistance in glioma (A,B) The mRNA and protein levels of UBE2C were greater in U-118 MG/TMZ (A) and LN-18/TMZ (B) cells than in U-118 MG and LN-18 cells, as measured by qRT-PCR and western blot analysis, *P<0.05. (C,D) CCK-8 assays were carried out to assess cell viability in U-118 MG/TMZ and LN-18/TMZ cells in the control, TMZ+OE-NC, TMZ+OE-UBE2C, TMZ-sh-NC, and TMZ+sh-UBE2C groups. (E‒G) Flow cytometry was performed to assess the apoptotic rates of U-118 MG/TMZ and LN-18/TMZ cells in the control, TMZ+OE-NC, TMZ+OE-UBE2C, TMZ-sh-NC, and TMZ+sh-UBE2C groups. (H,I) TUNEL staining was applied to U-118 MG/TMZ and LN-18/TMZ cells from the control, TMZ+OE-NC, TMZ+OE-UBE2C, TMZ-sh-NC, and TMZ+sh-UBE2C groups to assess cell apoptosis. (J,K) Western blot analysis was carried out to test the protein levels of UBE2C, Bcl-2, Bax, cleaved caspase-3 and caspase-3 in the U-118 MG/TMZ and LN-18/TMZ control, TMZ+OE-NC, TMZ+OE-UBE2C, TMZ-sh-NC, and TMZ+sh-UBE2C groups. *P<0.05 vs control group; #P<0.05 vs TMZ+OE-NC group; +P<0.05, vs TMZ+sh-NC group.

Then, we explored the role of UBE2C in regulating cell resistance to TMZ using *in vitro* experiments in glioma. First, we constructed TMZ-resistant cell lines (U-118 MG/TMZ and LN-18/TMZ), which had higher TMZ IC _50_ values than sensitive cells. Specifically, the IC _50_ values for U-118 MG/TMZ and U-118 MG cells were 174.6 μg/mL and 55.7 μg/mL, respectively ( Supplementary Figure S1A), while they were 160.8 μg/mL and 58.2 μg/mL for LN-18/TMZ and LN-18 cells, respectively ( Supplementary Figure S1B). In addition, we found that the mRNA and protein levels of UBE2C were approximately 1.8-fold and 0.9-fold greater, respectively, in TMZ-resistant U-118 MG/TMZ cells than in parental cells ( Figure 2A) and 1.3-fold and 0.8-fold greater, respectively, in LN-18/TMZ cells ( Figure 2B). Compared to those in the control group, TMZ exposure significantly suppressed U-118 MG/TMZ and LN-18/TMZ cell growth by approximately 50% at 48 h, which was further inhibited by UBE2C downregulation but restored following UBE2C upregulation ( Figure 2C,D). Moreover, we explored the role of UBE2C in regulating cell apoptosis in the presence of TMZ. The percentage of apoptotic U-118 MG/TMZ cells increased from approximately 5% to 13% following TMZ treatment, while the percentage of apoptotic U-118 MG/TMZ cells further increased to 30% when UBE2C expression was downregulated ( Figure 2E,F). A similar phenomenon was observed in the LN-18/TMZ cell line, whereas UBE2C overexpression reduced TMZ-induced apoptosis in both the U-118 MG/TMZ and LN-18/TMZ cell lines, as detected by flow cytometry ( Figure 2E‒G) and TUNEL ( Figure 2H,I) assays. Consistently, TMZ treatment increased the ratio of Bax expression to Bcl-2 expression, as well as the ratio of cleaved caspase-3 expression to caspase-3 expression, in both U-118 MG/TMZ and LN-18/TMZ cells, which was further enhanced by *UBE2C* silencing and suppressed by UBE2C overexpression ( Figure 2J,K). These results suggested that UBE2C triggered TMZ resistance in gliomas.

### UBE2C decreases p53 expression and promotes aerobic glycolysis in glioma

**Figure 3:**
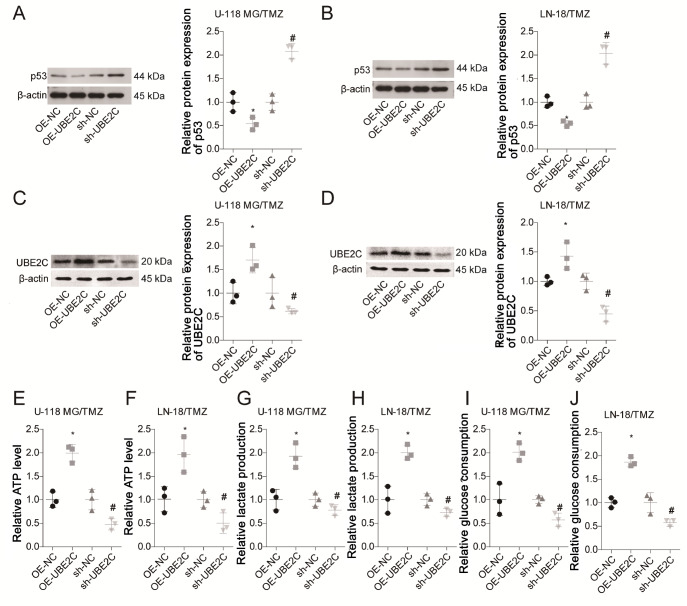
Figure 3 UBE2C decreases p53 expression and promotes aerobic glycolysis in glioma (A,B) The expression of UBE2C in U-118 MG/TMZ and LN-18/TMZ cells in the OE-NC, OE-UBE2C, sh-NC and sh-UBE2C groups was detected by western blot analysis. (C,D) p53 expression levels in the OE-NC, OE-UBE2C, sh-NC and sh-UBE2C groups of U-118 MG/TMZ and LN-18/TMZ cells were determined by western blot analysis. (E‒J) ATP levels, lactate production and glucose consumption in the OE-NC, OE-UBE2C, sh-NC and sh-UBE2C groups of U-118 MG/TMZ and LN-18/TMZ cells were tested using the corresponding kits. *P<0.05, vs OE-NC group; #P<0.05 vs sh-NC group.

Next, we explored the underlying mechanisms through which UBE2C triggers drug resistance to TMZ in gliomas. To this end, *UBE2C* was overexpressed or knocked down in both the U-118 MG/TMZ and LN-18/TMZ cell lines. UBC2C expression was significantly increased following cell transfection with OE-UBE2C, with overexpression rates of 70% and 40% in the U-118 MG/TMZ and LN-18/TMZ cell lines, respectively ( Figure 3A,B). In contrast, UBC2C expression was significantly decreased following cell transfection with sh-UBE2C, with knockdown rates of 45% and 62% in the U-118 MG/TMZ and LN-18/TMZ cell lines, respectively ( Figure 3A,B). Compared to that in the control group, the expression of p53 was reduced by approximately 50% when UBE2C was overexpressed in both the U-118 MG/TMZ and LN-18/TMZ cell lines, whereas silencing of *UBE2C* significantly increased p53 expression by 1.1-fold and 1-fold, respectively, compared to that in the control group ( Figure 3C,D). In addition, we found that ATP level ( Figure 3E,F), lactate production ( Figure 3G,H), and glucose consumption ( Figure 3I, 3J) were significantly elevated by approximately 1.7‒2.1-fold when UBE2C was overexpressed, whereas these levels decreased by approximately 0.4‒0.6-fold when UBE2C expression was downregulated in both the U-118 MG/TMZ and LN-18/TMZ cell lines. These results demonstrated that UBE2C decreased p53 expression and promoted aerobic glycolysis in gliomas.

### Knockdown of *UBE2C* sensitizes glioma cells to TMZ through increasing p53 expression

**Figure 4:**
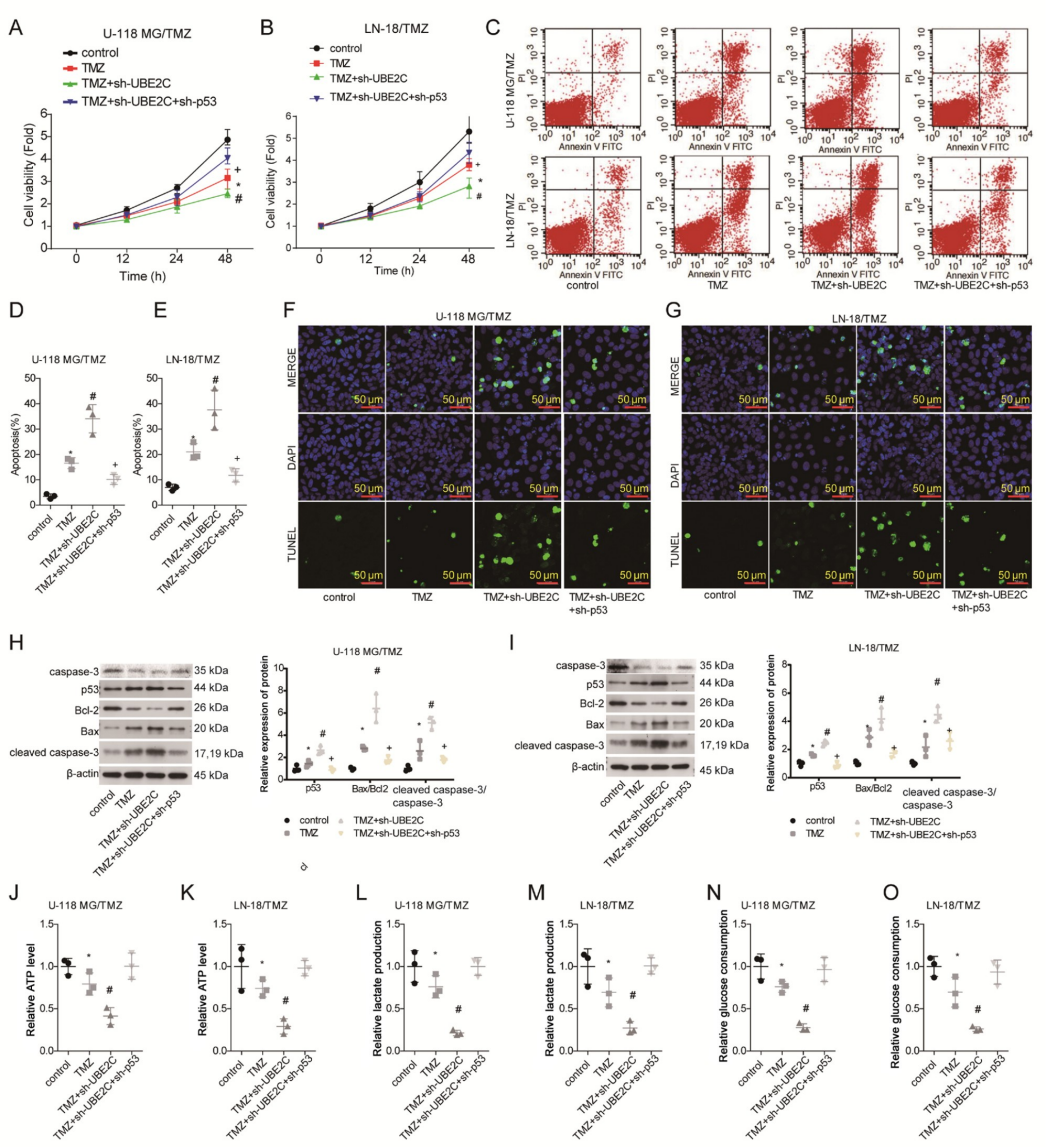
Figure 4 Downregulation of p53 weakens the TMZ sensitivity mediated by UBE2C downregulation in glioma U-118 MG/TMZ and LN-18/TMZ cells were divided into control, TMZ, TMZ+sh-UBE2C and TMZ+sh-UBE2C+sh-p53 groups, and the cells in each group were harvested for the following experiments. (A,B) Cell viability was tested by a CCK-8 assay. (C‒G) The cell apoptotic rate was measured by flow cytometry (C‒E) and TUNEL staining (F,G). (H,I) The protein levels of p53, Bcl-2, Bax, cleaved caspase-3 and caspase-3 were determined by western blot analysis. (J‒O) The levels of ATP (J,K), lactate production (L,M) and glucose consumption (N,O) were measured using the corresponding kits. *P<0.05 vs control group; #P<0.05 vs TMZ group; +P<0.05 vs TMZ+sh-UBE2C group.

Subsequently, we investigated whether p53 participates in UBE2C-mediated TMZ resistance in gliomas using rescue experiments. To this end, U-118 MG/TMZ and LN-18/TMZ cells were infected with sh-p53 and sh-UBE2C simultaneously or with sh-UBE2C only. The results demonstrated that p53 downregulation significantly impaired the ability of sh-UBE2C to inhibit cell viability in TMZ-treated U-118 MG/TMZ and LN-18/TMZ cells, with cell viability increased by approximately 80% ( Figure 4A,B). In addition, the cell apoptosis rate decreased by approximately 60%, as detected by flow cytometry ( Figure 4C‒E) and TUNEL ( Figure 4F,G) assays. To further confirm these findings, a western blot analysis was also performed to detect the expressions of apoptosis-related proteins. Similarly, the protein expression ratios of Bax/Bcl-2 and cleaved caspase-3/caspase-3 were decreased by approximately 70% and 50%, respectively, in cells infected with sh-p53 and sh-UBE2C at the same time compared to those in cells infected with sh-UBE2C alone ( Figure 4H,I). Moreover, downregulation of p53 and UBE2C simultaneously induced a 2-fold increase in ATP level ( Figure 4J,K), a 2.5-fold increase in lactate production ( Figure 4L,M), and a 1-fold increase in glucose consumption ( Figure 4N,O) in both the U-118 MG/TMZ and LN-18/TMZ cell lines. These results indicated that downregulation of UBE2C may sensitize glioma cells to TMZ through increasing p53 expression.

### Downregulation of UBE2C sensitizes glioma cells to TMZ *in vivo*

**Figure 5:**
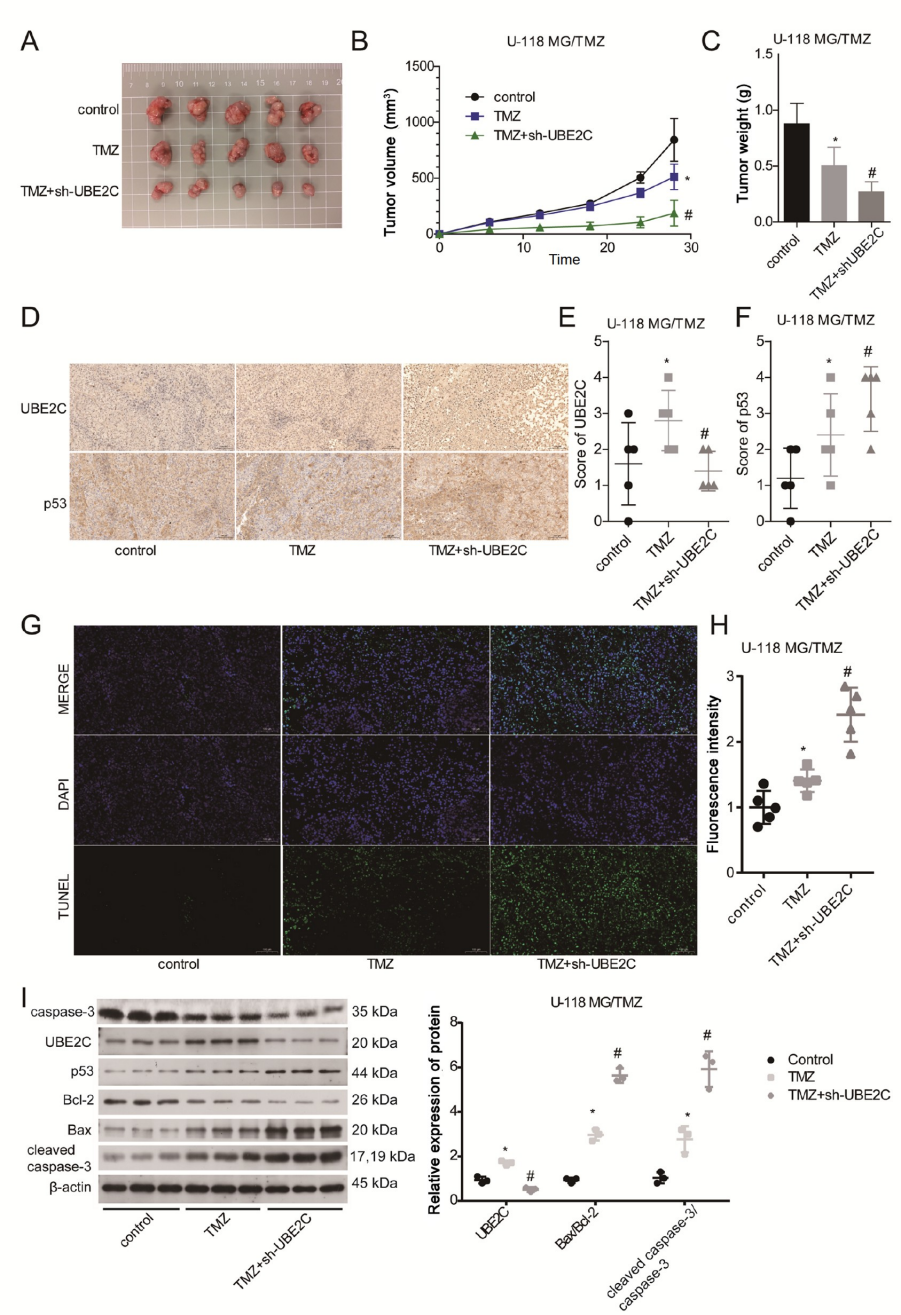
Figure 5 Downregulation of UBE2C sensitizes glioma cells to TMZ *in vivo* (A) Tumor morphology of U-118 MG/TMZ cells in the control, TMZ and TMZ+sh-UBE2C groups. (B,C) Tumor volume and weight of U-118 MG/TMZ cells in the control, TMZ and TMZ+sh-UBE2C groups. (D‒F) IHC staining of UBE2C and p53 in cancer tissues from U-118 MG/TMZ mice in the control, TMZ and TMZ+sh-UBE2C groups. (G,H) TUNEL was applied to assess cell apoptosis in cancer tissues from mice in the control, TMZ and TMZ+sh-UBE2C groups. (I) The protein levels of UBE2C, p53, Bcl-2, Bax, cleaved caspase-3 and caspase-3 in cancer tissues from mice in the control, TMZ and TMZ+sh-UBE2C groups were determined by western blot analysis. *P<0.05 vs control group; #P<0.05 vs TMZ group.

Finally, we investigated the role of UBE2C in modulating TMZ resistance in glioma *in vivo*. Compared with those in the TMZ group, the tumor volume and weight were decreased by approximately 50% following sh-UBE2C administration ( Figure 5A‒C). In addition, we observed an obvious decrease in the protein expression level of UBE2C and an increase in the expression level of p53 in the TMZ+sh-UBE2C group compared to those in the TMZ group, as detected by IHC staining ( Figure 5D‒F). In addition, we assessed cell apoptosis rates in glioma tissues from the three groups (control, TMZ, and TMZ+sh-UBE2C) using TUNEL and western blot analysis. The number of apoptotic cells (green staining) was markedly increased in the TMZ group, and it was further increased in the TMZ+sh-UBE2C group ( Figure 5G,H), as were the ratios of Bax/Bcl-2 and cleaved caspase-3/caspase-3 ( Figure 5I). Collectively, the *in vivo* assay further demonstrated that downregulation of UBE2C sensitized glioma cells to TMZ *in vivo*.

## Discussion

Arya *et al*. [18] analyzed the gene expression profile of xenografts from bevacizumab-resistant glioblastoma multiforme (GBM) patients and found that several hub genes, including *UBE2C*, were associated with GBM resistance to bevacizumab, suggesting that UBE2C might participate in regulating the drug resistance of glioma. However, the specific role of UBE2C in TMZ resistance in glioma and the underlying mechanisms remain unknown. In the present study, we showed that UBE2C was overexpressed in TMZ-resistant glioma tissues and cells and significantly enhanced TMZ resistance by downregulating p53 expression to increase aerobic glycolysis.

It has been demonstrated that UBE2C plays a vital role in regulating the drug resistance of several types of cancers, such as cisplatin resistance in ovarian cancer [6] and masitinib resistance in esophageal cancer [19]. In glioma, researchers have reported that *UBE2C* is highly expressed and functions as an oncogene; moreover, high expression of UBE2C is associated with poor prognosis in glioma patients [ 10, 11, 20, 21]. Consistently, we found that UBE2C was more highly expressed in glioma tissues than in normal brain tissues, and its high expression was linked to a lower overall survival rate in patients with gliomas. Additionally, it has been reported that UBE2C is implicated in drug resistance in gliomas. For instance, Alafate *et al*. [11] reported that the upregulation of AURKB and UBE2C led to unfavourable outcomes, such as shorter overall survival and therapy resistance, in glioma patients. Arya *et al*. [18] reported that *UBE2C* is one of several hub genes associated with GBM resistance to bevacizumab. Herein, we demonstrated that UBE2C was more highly expressed in both TMZ-resistant tissues and cells than in TMZ-sensitive tissues and parental cells. Moreover, we found that overexpression of UBE2C conferred TMZ-resistant cells with TMZ-mediated cell apoptosis and enhanced cell growth, whereas the downregulation of UBE2C impaired the antitumor effect of TMZ in glioma *in vitro* and *in vivo*. These results revealed that UBE2C functions as an inducer of TMZ resistance in gliomas.

Next, we investigated the mechanisms underlying UBE2C-mediated TMZ resistance in glioma. Aerobic glycolysis, the well-known “Warburg effect”, is a remarkable feature of cancer cells, even under aerobic conditions, in which glucose is converted into lactic acid [22]. A growing number of studies have revealed that aerobic glycolysis is strongly implicated in multiple tumorigenesis processes, including drug resistance [ 23‒ 26], providing a promising target for overcoming drug resistance in human tumors. Zhang *et al*. [27] revealed that the overexpression of mitochondrial Clk1 impaired chemoresistance through the AMPK/mTOR/HIF-1α-mediated glycolytic pathway in glioma cells. Yang *et al*. [28] reported that UBE2C could trigger HIF-1alpha-glycolytic flux in head and neck squamous cell carcinomas, suggesting a close link between UBE2C and aerobic glycolysis. In the present study, we also explored the role of UBE2C in modulating aerobic glycolysis in gliomas *in vitro*. As expected, ATP level, lactate production, and glucose consumption were significantly elevated in TMZ-treated U-118 MG/TMZ and LN-18/TMZ cells after UBE2C overexpression. These results demonstrated that UBE2C facilitated TMZ resistance in glioma possibly through enhancing aerobic glycolysis.

The tumor suppressor gene *TP53* plays crucial roles in the occurrence and progression of human tumors [29]. The p53 protein functions primarily as a transcription factor that modulates multiple biological processes, such as cell cycle arrest, DNA repair, autophagy, cell apoptosis, and metabolism, and determines whether cells die under stress conditions [ 30, 31]. In response to cellular stress, p53 prevents the differentiation of cells with mutated or damaged DNA and terminates cellular processes by transcriptionally regulating different genes involved in cell apoptosis and cell cycle [32], which contributes to its tumor suppressor ability, which is the most studied [ 33‒ 35]. In addition, many studies have revealed that p53 can also modulate a number of “nonclassical” pathways, including metabolic homeostasis, ferroptosis, stem cell differentiation, autophagy, senescence, and the tumor microenvironment [ 36‒ 39]. Furthermore, it has been demonstrated that p53 is a substrate of UBE2C; specifically, UBE2C facilitates p53 protein degradation in a ubiquitination-dependent manner in endometrial cancer cells [16]. Additionally, p53 has been reported to be involved in aerobic glycolysis in several types of cancers, such as hepatocellular carcinoma [ 40, 41], breast cancer [ 42, 43] and colon cancer [44]. Thus, we hypothesized that UBE2C may enhance TMZ resistance in glioma cells by downregulating p53 to trigger aerobic glycolysis. As expected, silencing of *p53* significantly impaired the ability of UBE2C downregulation to inhibit cell viability and aerobic glycolysis and markedly prevented cell apoptosis in TMZ-treated U-118 MG/TMZ cells and LN-18/TMZ cells. These results verified the hypothesis that UBE2C enhances TMZ resistance by regulating the p53/aerobic glycolysis axis in glioma cells.

In addition, alterations in the *TP53* gene are reported in approximately 25-30% of primary GBMs [45] with increased onset of *TP53* missense mutations, leading to disruption of the wild-type activity of p53 and its antitumor role [ 46, 47]. In this study, we found that the IC _50_ of the TP53-mutant LN-18 cell line was slightly greater than that of the TP53 wild-type U-118 MG cell line ( Supplementary Figure S1). In addition, overexpression of UBE2C significantly decreased the expression of p53 in both the TP53-mutant LN-18 cell line and the TP53 wild-type U-118 MG cell line, leading to resistance to TMZ. Conversely, downregulation of UBE2C rescued p53 expression and sensitized these two cell lines to TMZ regardless of the status of TP53, indicating that UBE2C enhances TMZ resistance by regulating the expression of p53 to induce aerobic glycolysis in glioma regardless of the mutational status of *TP53* in tumor cells.

In summary, this study demonstrated that UBE2C is overexpressed in TMZ-resistant glioma tissues and cells and that the overexpression of UBE2C significantly enhances the resistance of glioma cells to TMZ by limiting p53 expression and facilitating aerobic glycolysis. Overall, this study further clarified the mechanisms underlying TMZ resistance in gliomas, which may provide a potential therapeutic target for glioma patients with TMZ resistance.

## Supporting information

23566Supplementary_Figure_S1

## Supplementary Data

Supplementary data is available at *Acta Biochimica et Biophysica Sinica* online.
