# Supplementary material for: UBE2C enhances temozolomide resistance by regulating the expression of p53 to induce aerobic glycolysis in glioma: UBE2C enhances the TMZ resistance in glioma
Source: Acta Biochim Biophys Sin (Shanghai). 2024 Apr 17;56(6):916–26. doi: 10.3724/abbs.2024033 (PMC11214954; doi:10.3724/abbs.2024033)
Supplement: 23566Supplementary_Figure_S1 [file 23566Supplementary_Figure_S1.pdf]

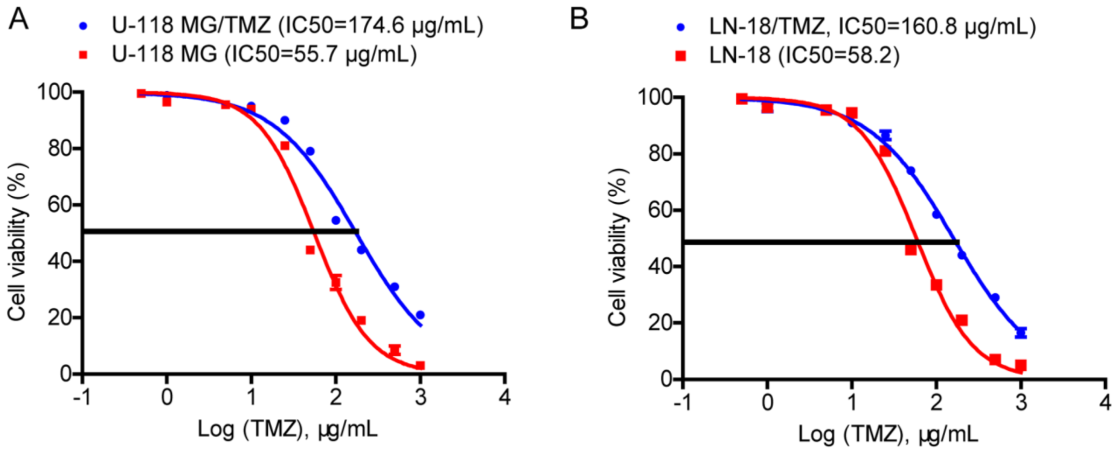

Supplementary Figure S1. IC$_{50}$ of TMZ in the TMZ-resistant and parental cell lines A CCK-8 assay was used to assess the IC$_{50}$ values of (A) U-118 MG/TMZ and U-118 MG cells and (B) LN-18 MG/TMZ and LN-18 MG cells.
